# Urticaria and infections

**DOI:** 10.1186/1710-1492-5-10

**Published:** 2009-12-01

**Authors:** Bettina Wedi, Ulrike Raap, Dorothea Wieczorek, Alexander Kapp

**Affiliations:** 1 Department of Dermatology and Allergy, Hannover Medical School, Hannover, Germany

## Abstract

Urticaria is a group of diseases that share a distinct skin reaction pattern. Triggering of urticaria by infections has been discussed for many years but the exact role and pathogenesis of mast cell activation by infectious processes is unclear. In spontaneous acute urticaria there is no doubt for a causal relationship to infections and all chronic urticaria must have started as acute. Whereas in physical or distinct urticaria subtypes the evidence for infections is sparse, remission of annoying spontaneous chronic urticaria has been reported after successful treatment of persistent infections. Current summarizing available studies that evaluated the course of the chronic urticaria after proven Helicobacter eradication demonstrate a statistically significant benefit compared to untreated patients or Helicobacter-negative controls without urticaria (p < 0.001). Since infections can be easily treated some diagnostic procedures should be included in the routine work-up, especially the search for Helicobacter pylori. This review will update the reader regarding the role of infections in different urticaria subtypes.

## Introduction

Urticaria is a group of disorders that share a distinct skin reaction pattern, namely the occurrence of itchy wheals anywhere on the skin. Wheals are short-lived elevated erythematous lesions ranging from a few millimetres to several centimetres in diameter and can become confluent. The itching can be pricking or burning and is usually worse in the evening or night time [1]. Nearly half of the patients report sleep disturbances [2]. Typically, the lesions are rubbed and not scratched; therefore, usually excoriated skin is not a consequence of urticaria. Itchy wheals and also angioedema, which occur in about half of the patients from time to time, are the result of the degranulation of mast cells and basophils with release of mediators, predominantly histamine. Possible direct and indirect mechanisms by which degranulation is induced include autoreactivity including autoimmunity mediated by functional autoantibodies directed against the high affinity IgE receptor or IgE, infections (e. g. with Helicobacter pylori), non-allergic hypersensitivity reactions (e. g. to acetylsalicylic acid) and others such as internal diseases/malignancies [3]. The considerable variation in the frequency of underlying causes in different studies might reflect differences in patient selection.

In long-persisting urticarias the diagnostic work-up is dependent on clues identified by history. The treatment is removal of specific and non-specific triggers and the use of symptomatic medications attenuating mediator effects such as non-sedating H1-antihistamines.

Based upon duration and eliciting factors three main urticaria subgroups should be differentiated: i) spontaneous urticaria, ii) physical urticaria, iii) other special forms. Most common is spontaneous urticaria which is defined to be acute if the whealing persists for less than six weeks and chronic if it persists longer. At least half of the patients suffer from additional recurrent angioedema that are considered to be histamine-mediated but are located deeper in skin than wheals. Up to 15% of patients have recurrent angioedema without wheals and flares and with normal C1-Esterase inhibitor (in contrast to hereditary C1-Esterase-Inhibitor deficient angioedema). Physical urticaria types are triggered by exogenous factors such as cold, pressure, heat, vibration. Other distinct forms are cholinergic urticaria (triggered by increase of body temperature), aquagenic urticaria (triggered by water), contact urticaria (triggered by contact to urticariogenic substance) or exercise-induced urticaria. Two or more urticaria subtypes can coexist in any given patient.

Except in acute urticaria, most forms are highly chronic and persist often for several years to decades [4,5]. Quality of life is significantly impaired [6-8].

A role of infections in urticaria subtypes is discussed for more than 100 years and has been included in most reviews. As early as in the 1920ies it was thought that "in a large majority of chronic urticaria the origin of the trouble is to be found in such septic centres" [9]. Bacterial infections of the teeth, the tonsils, e.g. with streptococci, staphylococci had been described [10]. Nevertheless, the exact role and pathogenesis of mast cell activation by infectious processes remains unclear [7]. A causal relation with underlying or precipitating infection is difficult to establish, since there is no possibility to challenge the patient with the suspected pathogen.

Looking at current international or national urticaria guidelines different recommendations can be found:

For example the British Guideline from 2007 states: „...Associations between chronic urticaria and occult infection (e.g. dental abscess and gastrointestinal candidiasis) have been proposed but there is little evidence to support them (Quality of evidence III). A meta-analysis of therapeutic trials for Helicobacter pylori found that resolution of chronic urticaria was more likely when antibiotic therapy was successful than when it was not (Quality of evidence I, Strength of recommendation B)” and „... infections may play a causative role in a few cases, and when present, chronic infections such as dental sepsis, sinusitis, urinary tract infections and cutaneous fungal infections should be treated. However exhaustive investigations searching for underlying infections are not indicated. Infection with Helicobacter pylori (HP) has been proposed as a possible cause, but the association is unlikely to be causal. Candida colonization of the gut is not a cause of chronic urticaria”.

The European Guideline from 2006, that is currently updated, states: "Among others, chronic infections (e.g. Helicobacter pylori), nonallergic intolerance reactions to foods and autoreactivity functional autoantibodies directed against the immunoglobulin E (IgE) receptor have been described. However, there is considerable variation in the frequency of eliciting factors in the different studies."

### Role of infections in spontaneous acute urticaria with/without angioedema

In most cases spontaneous acute urticaria disappears within two to four weeks (by definition within six weeks).

Acute urticaria is considered as a classical manifestation of viral infection in general, especially in children [7,11,12] but also in adults. Recent data found infections to be the most common identified cause (37%) [13]. Accordingly, in children infections were identified in 57% of acute urticaria cases [14], benign viral upper respiratory (nasopharnygitis) or digestive infections being the most frequent etiology.

Due to the limited duration of spontaneous acute urticaria systematic studies are rare. With regard to general inflammatory parameters leukocytosis was found in 36/57 children (63%) with acute urticaria and elevated C-reactive protein in 16/36 (44%) [15].

Reports described acute urticaria caused by streptococcus, mycoplasma pneumoniae, parvovirus B19, norovirus, enterovirus, Hepatitis A or B (so called "yellow" urticaria), and plasmodium falciparum (Table 1). A review [16] summarized five studies and found upper respiratory viral infections to affect about half of the patients with acute urticaria. Seasonal variations of incidence have been explained by peaks of infections with influenza-, adeno-, respiratory syncytial, possibly also parainfluenza and rhinoviruses in winter and different types of coxsackie-, corona- and adenoviruses in June [17]. In addition, acute urticaria due to influenza vaccination has been reported [18].

**Table 1:** Number of reported infections in different urticaria subtypes published in PubMed (01.02.2009)

Pathogen	acute UR	chronic UR	cold UR	DPU	AE (histam.)	AAE	HAE
**Bacteria**							
H. pylori	nd	+++	1	nd	+	+	++
Streptococcus spp.	+++	+++	nd	nd	+	nd	Nd
Staphylococcus spp.	++	++	nd	nd	nd	nd	Nd
Yersinia ent. (persist.)	nd	+++	nd	nd	nd	nd	Nd
Mycoplasma pneum.	+ (c)	nd	+	nd	nd	nd	Nd
Trichinella	nd	1	nd	nd	nd	nd	Nd
Plasmodium falcip.	+	nd	nd	nd	nd	nd	Nd
**Viruses**							
HIV	nd	nd	+	nd	nd	nd	Nd
Influenza	+	nd	nd	nd	nd	nd	Nd
Adenovirus	+ (c)	nd	nd	nd	nd	nd	Nd
Enterovirus	+ (c)	nd	nd	nd	nd	nd	Nd
Rotavirus	+ (c)	nd	nd	nd	nd	nd	Nd
RSV	+ (c)	nd	nd	nd	nd	nd	Nd
Hepatitis A virus	+	nd	nd	nd	nd	nd	Nd
Hepatitis B virus	+	nd	+	nd	+	nd	Nd
Hepatitis C virus	nd	++	1	nd	nd	nd	Nd
Cytomegalovirus	+	+	+	nd	nd	nd	Nd
Epstein-Barr virus	+	+	nd	nd	+	nd	+
Parvovirus B19	+	nd	nd	nd	+	nd	Nd
Norovirus	1	1	nd	nd	nd	nd	Nd
**Parasites**							
Blastocystis hominis	+	++	nd	1	1	nd	Nd
Giardia lamblia	nd	+	nd	nd	1	nd	Nd
Toxocara canis	nd	+	1	nd	nd	nd	Nd
Trichomonas vagin.	nd	1	nd	nd	nd	nd	Nd
Anisakis simplex	++	nd	nd	nd	nd	nd	Nd
Strongyloides sterc.	nd	+	nd	nd	+	nd	Nd
**unspecified**							
rhinopharnygitis	+++	nd	nd	nd	++	nd	Nd
tonsillitis	nd	++	nd	nd	+	nd	Nd
sinusitis	nd	++	nd	nd	+	nd	Nd
dental infection	+	++	nd	nd	+	nd	Nd
urinary tract infection	++	++	nd	nd	+	nd	Nd

Number of publications:

1, if n = 1; +, if n = 2-10; ++, if n = 11-99; +++, if n ≥ 100; nd, not described

*Search strategy in PubMed: titles/abstract for the respective pathogen and "urticaria"

Items with "urticaria" in title/abstract: 8336

Abbreviations: UR, urticaria; DPU, delayed pressure urticaria; AE, angioedema; histam., histaminergic; AAE, acquired angioedema; HAE, hereditary angioedema; (c), children

However, bacterial infections such as cystitis and tonsillitis can be also found in association with acute urticaria [12,19,20]. Already 1964 it has been described that antistreptolysin titres are significantly more common in acute urticaria compared to controls [21].

20-30% of children with spontaneous acute urticaria, 91% of them caused by infection, progressed to chronic urticaria [12].

In southern countries acute urticaria might be caused by Anisakis simplex, a seafish nematode, after eating uncooked fish[22,23] However, the role of Anisakis in recurrent acute urticaria is discussed controversially [24-26]. Double blind placebo-controlled oral challenges with 100 lyophilized Anisakis simplex larvae, or its equivalent in antigen, did not induce clinical symptoms in individuals with a clinical history and laboratory findings of hypersensitivity to Anisakis simplex [27].

### Role of infections in spontaneous chronic urticaria with/without angioedema

Whereas the prevalence of infections, bacterial, viral, parasitic or fungal, appears not to differ in spontaneous chronic urticaria compared to the general population, there is a very large amount of reports demonstrating benefit after eradication of infectious processes [7].

Published data differ substantially with respect to study population, diagnostic tests, evaluation procedure and follow-ups, assessment of treatment and interpretation. Moreover, often the multitude of triggering factors has been oversimplified.

Regarding general inflammatory parameters elevated erythrocyte sedimentation was found in 2% of 66 patients with chronic urticaria, abnormal C-reactive protein in 16% of 88 patients, and leukocytosis in 23% of 133 patients [28].

Most reported infections in chronic urticaria are related to the gastro-intestinal tract, but also to the dental or ENT region.

### Gastro-intestinal infections

Several gastro-intestinal infections have been linked to chronic urticaria (Table 1). Best evidence exists for Helicobacter pylori infection (Table 2).

**Table 2:** Pro-Studies evaluating Helicobacter pylori (HP) and chronic urticaria (CU), (only studies that included proven eradicated patients)

H. pylori proven eradication rate (%)	first treatment regimen (days)	evaluation period in months	Remission or improvement rate (%)			First author Country Year	**Ref**.
			eradicated HP+ CU	untreated HP+ CU	untreated HP- CU		
39/46 (85)	OAC (14)	3	36/39 (92)*	10/20 (50)	nd	Yadav India 2008	[37]
39/45 (87)	OAC (14)	2 and 4	nd**	nd**	0/33	Magen Israel 2007	[36]
62/68 (91)	OAC (7)$	nd	41/55 (75)	nd	nd	Vazquez Spain 2004	[101]
26/26 (100)	OAC (4)$	>12	19/26 (73)	nd	nd	Shiotani Japan 2001	[76]
24/24 (100)	OAC (7)$	4	19/21 (91)	10/20 (50)	33/53 (62)	Wedi Germany 1998	[52]
21/21 (100)	BM, ABM or BMT	not specified	20/21 (95)	nd	nd	Kolibasova Slovakia 1994	[102]
20/20 (100)	OAC (7)	not specified	14/20 (70)	nd	nd	Gaig Spain 2002	[103]
17/19 (89)	LAC, LAM (7)	2	17/17 (100), CR in 6/17	2/9 (22)	nd	Fukuda Japan 2004	[34]
17/17 (100)	ABM or OA (7)	not specified	17/17 (100)	nd	nd	Kalas Hungary 1996	[104]
16/18 (89)	LAC (7)	1-4	16/16 (100)	nd	0/19 (0)	Di Campli Italy 1998	[105]
15/15 (100)	LAC (7)	>6	9/15 (60)	nd	nd	Sakurane Japan 2002	[106]
14/17 (82)	OA, OC or OMT (7)	1.5-2.5	14/17 (82)	nd	0/8 (0)	Tebbe Germany 1996	[107]
12/12 (100)	OAM (7)	not specified	10/12 (83)	nd	nd	Bonamigo Brazil 1999	[108]

**Total** **322/348** **(93)**	**§**		**Total** **232/276** **(84)**^&^	**Total** **22/49** **(45)**	**Total** **33/113 (29)**		

*signif. increase of CU_2_QoL (p = 0.001)

**signif. Urticaria activity score decrease in eradicated HP+CU but not in untreated HP+CU or untreated HP-CU

$treatment was repeated or a reserve schedule was used until eradication was achieved

§9 of 13 studies (69%) used a triple treatment of proton pump inhibitor (O or L) plus amoxicillin and clarithromycin;

^&^study by Magen could not be included because remission/improvement rates were not described

Abbreviations: O, omeprazole; A, amoxicillin; C, clarithromycin; B, bismuth; M, metronidazole; T, tetracycline; L, lansoprazole; HP, Helicobacter pylori; CU, chronic urticaria; nd, not done;

Systematically reviewing existing studies addressing the effect of antibiotic therapy for chronic urticaria patients infected with **Helicobacter pylori **in 2003 revealed that resolution of urticaria was more likely when antibiotic therapy was successful in eradication of Helicobacter [29]. 10 studies fulfilled the inclusion criteria and when data from these studies were combined, eradication of Helicobacter pylori was both significantly associated with remission of urticaria (odds ratio 2.9, 95% confidence interval 1.4-6.8; p = 0.005). When comparing the remission in patients with H. pylori eradication to H. pylori negative patients with chronic urticaria the odds ratio increased to 4.7 (95% CI 2.6-17.6, p = 0.001) [29]. The authors concluded that clinicians, after considering other causes of urticaria, should constitute (1) testing for Helicobacter pylori; (2) treating with appropriate antibiotics if Helicobacter pylori is present; and (3) confirming successful eradication of infection [29]. We are recommending this approach for several years [7,19,30-32].

The problem of several studies, including one from 2008 [33] that argue against a benefit of Helicobacter pylori eradication in chronic urticaria, is, that a control of the eradication status was missing or inadequate eradication schedules or control methods were used. This has been reviewed in detail [1]. Control of eradication success is of particular importance since the success of eradication with triple therapy has been decreasing with increasing resistance to commonly used antimicrobials. A multicenter study published in 2001 [4] demonstrated significant resistance rates against metronidazole and clarithromycin in Western countries of up to 62% (Helsinki, Finland) and 27% (Chieti, Italy), respectively (for amoxicillin up to 8%) [4]. In this regard, clinicians should bear in mind that an urea breath test or a Helicobacter pylori stool antigen test can be false negative (e.g. if proton pump inhibitors are not stopped four weeks before).

After publication of the systematic review in 2003 [29] several studies from different countries (Japan, Mexico, Israel, India) have confirmed a clear and statistical significant benefit of Helicobacter eradication in chronic urticaria (Table 2) [34-37]. In all these studies eradication status was proven 4-6 weeks after the treatment and only patients with proven eradication of Helicobacter pylori were evaluated regarding the outcome of their urticaria. Moreover, in two studies well-accepted objective urticaria activity scores or standardized quality of life instruments were used to demonstrate that proven eradication of Helicobacter pylori resulted in a statistically significant reduction of urticaria activity score [36] and a significant increase of quality of life (CU-Q_2_ol) [37], respectively.

Nowadays, the combination of all studies regarding Helicobacter that included patients with chronic urticaria, excluded other causes of urticaria by appropriate testing, and controlled success of eradication treatment resulted in the identification of 13 studies (including a total of 322 eradicated patients) *pro *a benefit of Helicobacter eradication for chronic urticaria (Table 2) and 9 studies (including 164 eradicated patients) *contra *a benefit (Table 3). In the studies that apply for a benefit 84% of patients demonstrated significant improvement or complete remission of chronic urticaria after eradication of Helicobacter, in contrast to 45% of untreated Helicobacter positive, and 29% of untreated Helicobacter negative patients with chronic urticaria (Table 2). Looking at the treatment schedules it is interesting that most *pro *studies (69%) used a triple treatment consisting of a proton pump inhibitor plus amoxicillin plus clarithromycin, the two newer studies by Magen [36] and Yadav [37] even for two weeks, in contrast to only 44% of *contra *studies. Thus, future studies should clarify whether the type of treatment schedule has an impact on the urticaria remission rate.

**Table 3:** Contra-Studies evaluating Helicobacter pylori (HP) and chronic urticaria (CU) (only studies that included proven eradicated patients)

H. pylori proven eradication rate (%)	first treatment regimen (days)	evaluation period in months	Chronic urticaria remission or improvement rate (%)			First author Country Year	**Ref**.
			treated HP+ CU	untreated HP+ CU	untreated HP- CU		
29/31 (94)	OMC (7)	4-12	6/31 (19)	3/34 (9)	nd	Valsecchi Italy 1998	[109]
25/29 (86)	OAC (7)	nd	1/25 (4)	nd	nd	Erel Turkey 2000	[110]
24/30 (80)	OA (7)	6	8/30 (27)	nd	nd	Wustlich Germany 1999	[111]
17/23 (74)	OAC (7)	4-6	5/17 (29)	nd	3/8 (38)	Özkaya-Bayazit Turkey 1998	[112]
12/13 (92)	AM (7)	4	8/11 (73)	9/13 (69)	nd	Schrutka-Koelbel Austria 1998	[113]
12/15 (80)	OAC (7)	>12	3/12 (25)	nd	nd	Daudén Spain 2000	[114]
30/35 (86)*	LMA or LMT (7)	5	8/30 (27)	5/18 (28)	nd	Hook-Nikanne Finland 2000	[115]
3/11 (27)	LA (7)	3-6	1/3 (33)	4/14 (29)	nd	Schnyder Switzerland 1999	[116]
12/14 (80)	OAC (7)	6	3/12 (25)	nd	nd	Moreira Portugal 2003	[117]

**Total** **164/201** **(82)**	**§**		**Total** **43/171** **(25)**	**Total** **21/79** **(27)**	**Total** **3/8** **(38)**		

§ 4/9 (44) studies used a triple treatment of proton pump inhibitor (O or L) plus amoxicillin and clarithromycin

Abbreviations: O, omeprazole; A, amoxicillin; C, clarithromycin; B, bismuth; M, metronidazole; T, tetracycline; L, lansoprazole; HP, Helicobacter pylori; CU, chronic urticaria; nd, not done.

Taken all studies (*pro *and *contra*) together (Table 4), the rate of remission of urticaria when Helicobacter pylori was eradicated was 61.5% (275/447) compared with 33.6% (43/128) when Helicobacter pylori was not eradicated; the background remission rate among control subjects without H pylori infection was 29.7% (36/121). To account for the clustering of observations, a chi-square test was used. Compared to the review published in 2003, the difference is much clearer in favour of a benefit of Helicobacter eradication (p < 0.001) (Table 4). The remission/improvement rate is nearly doubled when Helicobacter pylori is eradicated.

**Table 4:** Combination of the chronic urticaria remission/improvement rates of all studies, namely pro (see Table 2) and contra (see Table 3) together (= this review 2009) and comparison to the systematic review of Federman [29]].

	Chronic urticaria remission or improvement rate (%)			
	eradicated HP+ CU	untreated HP+ CU	untreated HP- CU	p-value Chi-square
Federman	59/191 (30.9%)	18/83 (21.7%)	10/74 (13.5%)	p = 0.008
This review	275/447 (61.5%)	43/128 (33.6%)	36/121 (29.7%)	p < 0.001

Abbreviations: HP, Helicobacter pylori; CU, chronic urticaria

Concerning other gastro-intestinal infections, two studies described abnormal serology for yersinia (abnormal anti-yersinia-IgA and several bands in the immunoblot) consistent with **persistent yersiniosis **in 39% (36/93 chronic urticaria patients) and 31% (46/145 patients), respectively [38]. In several cases remission of urticaria after adequate antibiotic treatment (quinolones or TMP/SMX) was observed. In addition, a more recent study found specific antibodies for yersinia (IgG) in 31/74 (42%) of chronic urticaria patients [19,33].

Looking at **viral **gastrointestinal infections there is one report that linked chronic urticaria to infection with Norovirus [39]. In contrast, a review regarding a link between hepatitis viruses and chronic urticaria concluded that no convincing argument exists to suggest causality [40].

Regarding **parasites **there is evidence for a role of infestation with Blastocystis hominis and Giardia lamblia [41,42]. A study from Switzerland found parasites in stool in 35% of 46 patients with chronic urticaria, most of them with Blastocystis hominis [28]. More rarely, other parasites such as trichinella, trichomonas vaginalis, toxocara canis have been described (Table 1). However, occult parasitosis is not a likely cause in Western populations, and peripheral blood eosinophilia nearly always indicates infestations.

Regarding **fungi**, recent data found no evidence for an association of **Candida spp**. with chronic urticaria [43]. Intestinal and oral colonization of Candida spp. and serological evidence (ELISA anti-Candida-IgG/-IgM/-IgA) of Candida infections were not significantly different between patients with chronic urticaria (n = 38) and controls (n = 42) [43]. In contrast, anti-Candida-IgE was found in another study [44]. The meaning is unclear, particularly because the findings were associated with elevated total IgE and could therefore be non-specific. Actually, candidiasis is regarded not to play a significant role for chronic urticaria although it should be treated if the colonization is extensively [45].

### Dental or ENT focal infection

A study published in 1964 found that roentgenographic examinations found sinusitis in 32% of 59 chronic urticaria, and dental focal infections in 29% of 45 patients [20]. At least 8 cases with complete remission of chronic urticaria after elimination of dental focal infections have been described [46-52]. However, two studies did not find a significant association between chronic urticaria and dental infections [53,54].

A recent study identified tonsillitis or sinusitis in almost 50% of analyzed patients [55]. Anti-streptococcal antibodies have been described in 10-42% of patients with chronic urticaria, and antistaphylolysin antibodies in 1-10% of patients (reviewed in [7]).

Recent data found high nasal carriage of Staphylococcus aureus in patients with chronic urticaria compared to controls suggesting that nasal carriage functions as focus [56]. Moreover, a sub-population of patients with chronic urticaria was demonstrated to have serum IgE antibodies to SEA, SEB, and TSST [57].

In a study published in 1967 it was described that the outstanding historical feature in 15 of 16 children with chronic urticaria was recurrent upper respiratory infection, pharyngitis, tonsillitis, sinusitis, otitis, often by streptococci or staphylococci [58]. Remission of urticaria was frequently noted following antibiotic therapy [58]. This fits with our clinical experience in children [7,59].

Nevertheless, systematic antibiotic treatment studies of dental or ENT focal infections are lacking although benefit after oral cephalosporin or amoxicillin treatment has been described [7].

### Role of infections in angioedema without wheals

Angioedema is a self-limited nonpitting edema generally affecting the deeper layers of the skin and mucous membranes. It is the result of increased vascular permeability causing the leakage of fluid into the skin in response to potent vasodilators released by immunologic mediators. Two main pathways are thought to be implicated in angioedema. The most common is the mast cell pathway in which preformed mediators, such as histamine, leukotrienes and prostaglandins, are released from mast cells through IgE or direct activation. The second pathway is the kinin pathway, most notably affected by angiotensin converting enzyme (ACE) inhibitors and hereditary forms of angioedema with C1-esterase inhibitor defects, which ultimately results in the formation of bradykinin, a potent vasodilator.

Studies focusing on the role of infections in isolated recurrent angioedema are sparse. Regarding mast-cell mediated recurrent angioedema a recent study found 27 of 776 cases to be of infectious origin (e. g. dental granuloma, sinusitis, urinary tract infection) [60]. However, in this study in 41% (n = 315) the etiology remained unclear. Regarding infectious triggers the following investigations were performed routinely: blood cell count, erythrocyte sedimentation rate, C-reactive protein, hepatic enzymes, sinus and dental radiographs, stool examinations for ova and parasites and urine analyses, pharyngeal and urine samples were cultured [60]. This means that infections with Helicobacter pylori, streptococci, staphylococci, and yersinia could have been overlooked.

Among children, infection is regarded to be a common cause of angioedema [61]. Viral infections such as herpes simplex, coxsackie A and B, hepatitis B, Epstein-Barr, and other viral illnesses such as upper respiratory tract infections have been described. Bacterial infections associated with angioedema among children include otitis media, sinusitis, tonsillitis, upper respiratory tract infections, and urinary tract infections. Parasitic infections are considered to be less common, but may be possible with strongyloides, toxocara, and filarial [61].

An exacerbating role of Helicobacter pylori infection in hereditary angioedema is well established [62-64]. These authors stated that, whatever the mechanism of the association between H. pylori infection and HAE attacks, screening for H. pylori infection and eradication of this pathogen from infected patients with frequently recurring abdominal symptoms both seem warranted.

In addition, two cases with acquired C1-Esterase-Inhibitor deficiency due to autoantibodies have been associated with Helicobacter pylori infection [65,66].

### Physical types of urticaria and infections

Physical urticaria accounts for about 15% of urticaria cases and is triggered by exogenous mechanical stimuli that by both, immunologic and non-immunologic mechanisms - cause mast cell activation. Often these physical urticaria subtypes persist for average 3-5 years and may interfere with ability to work. Recent data demonstrated that also in children physical urticaria is long-persisting: only 38% were symptom-free 5 years post-onset [67].

Physical urticaria is divided into several subtypes with dermatographic urticaria (urticaria factitia) being the commonest form, followed by cold urticaria and delayed pressure urticaria whereas heat urticaria, solar and vibratory urticaria are very rare. In physical urticaria the routine diagnosis is mainly limited to identify the subtype by appropriate standardized challenge tests [68].

The association of physical urticaria to infections has been poorly studied. Most physical urticarias are regarded not to be associated with infection but systematic studies are lacking and often no investigations regarding infections have been performed.

There are no reports of infections in **dermographic urticaria **(urticaria factitia) although it is generally accepted that dermographism can start after infections and/or drug intake (e. g. penicillin).

The vast majority of cold urticaria is idiopathic but up to 5% of **cold urticaria **is regarded to be associated with infections (syphilis, borreliosis, measles, varicella, hepatitis, mononucleosis, HIV). However, a recent analysis of 62 patients with cold urticaria did not find relevant underlying infections (investigating hepatitis profile, serology for EBV, syphilis, and stool analysis for parasitosis) [69]. Nevertheless, in an open study 20-50% of patients responded to antibiotic treatment pointing to the possibility of unrecognized bacterial infections [70]. One case of acquired cold urticaria was cured after eradication of Helicobacter pylori [71].

### Other types of urticaria and infections

**Cholinergic urticaria **caused by increased body temperature after physical exercise and/or emotional stress is common in young adults. The long presumed hypothesis that it may be caused by sensitization to sweat antigens is under investigation [72].

There are no reports dealing with infections in cholinergic urticaria.

The pathogenesis of **aquagenic urticaria **remains unclear, although there is evidence to suggest that it is histamine-mediated. A single case of aquagenic urticaria associated with HIV infection has been described [73].

### Pathogenetic mechanisms

The pathogenetic mechanisms by which infections may induce urticaria are far from being clear. Several hypotheses have been developed, particularly regarding the link with Helicobacter pylori. There is increasing evidence for systemic effects of gastric Helicobacter pylori infection, which may be involved in extra-gastrointestinal disorders such as vascular, autoimmune and skin diseases.

Interestingly enough, actually, in patients with chronic idiopathic thrombocytopenic purpura (ITP) and Helicobacter pylori infection eradication is suggested by the European Helicobacter Study Group consensus 2007 [74].

In chronic urticaria several studies have described specific immune responses to Helicobacter pylori infection.

For example, the prevalence of IgA- and IgG-antibodies to Helicobacter pylori-associated lpp20 antibodies was significantly higher in Helicobacter pylori-infected chronic urticaria compared to the control group of patients with severe H. pylori-associated gastritis without urticaria (93.9 vs. 21.2%, P < 0.0001 for IgG, and 46.1 vs. 6.3%, P < 0.0029 for IgA) [75].

In single cases specific IgE antibodies to Helicobacter antigens have been described. For example, an IgE antibody to a 44 K antigen was found in 2/2 patients with chronic urticaria and complete remission after Helicobacter eradication [76]. In a similar case Gala-Ortiz et al. [77] demonstrated IgE binding to a Helicobacter pylori antigen by immunoblotting. Moreover, Mini et al. demonstrated a specific IgA- and IgE-mediated immune response against antioxidative bacterial proteins in chronic urticaria patients but not in Helicobacter-infected dyspeptic patients without urticaria [78].

Host factors like the presence in gastric mucosa of Lewis b antigen, a receptor for Helicobacter pylori, could be an individual susceptibility factor for infection and indirectly for development of other symptoms related to the antimicrobial immune response. It is tempting to speculate that structural components like adhesins or products of Helicobacter pylori such as urease, protease, phospholipase or cytotoxins may be released and may trigger complement activation.

Significantly increased gastric juice ECP (eosinophil cationic protein) and gastric eosinophil infiltration were described in Helicobacter pylori-infected chronic urticaria compared to both uninfected chronic urticaria patients and normal controls [79]. Moreover, Helicobacter pylori eradication resulted in a significant decrease in gastric juice ECP and gastric eosinophil infiltration only in chronic urticaria patients.

The expression of two H. pylori proteins, cytotoxin associated protein (cag A) and vacuolization cytotoxin (vac A) is considered to be related with pathogenicity of the bacterium. Infection with cag A+ strains is more common in patients with acne rosacea, stroke and coronary heart disease. Only few studies investigated cag A or vac A status in Helicobacter-infected chronic urticaria patients: Shiotani et al. did not find any distinctive features investigating the seroprevalence of IgG and IgE antibody against cag A and vac A [76]. Fukuda et al. found the cag A gene in all Helicobacter pylori strains isolated from the gastric mucosa of 13 patients with chronic urticaria, but this was comparable to Helicobacter-positive controls without urticaria [34].

### Infection and autoimmunity/autoreactivity

Spontaneous chronic urticaria is associated with autoreactivity/autoimmunity in at least one third of patients. Details of the autoimmune pathogenesis of chronic urticaria have been reviewed elsewhere [80-82].

Particularly autoantibodies to thyroid and histamine releasing autoantibodies to the high affinity IgE receptor (FcεRI) or to IgE are found. Moreover, the serum of patients with positive autologous serum skin test contains undefined immunoglobulin-independent stimulators of mast cell/basophil activation resulting not only in histamine release, but also in cysteinyl leukotrienes production and basophil activation (CD63 surface expression) [83]. In this regard, complement mediated augmentation of mast cell/basophil degranulation or activation may play a major role but does not explain all the functional effects [84-86].

In addition, the association of certain HLA class II alleles supports an autoimmune pathogenesis in a subset of patients with chronic urticaria [87-89].

Autoreactivity occurs when the immune system recognizes host tissue and infectious pathogens such as bacteria are thought to play a major role in its development [90]. Mechanisms by which pathogens might cause autoimmunity include (a) molecular mimicry (pathogen-derived epitopes cross-react with self-derived epitopes); (b) epitope spreading (the persisting pathogen causes damage to self-tissue by inducing direct lysis or immune response), (c) bystander activation (non-specific activation of various parts of the immune system), or (d) cryptic antigens (subdominant cryptic antigens appear after inflammatory reactions) [90].

Regarding the pathogens that have been described in spontaneous chronic urticaria some of these mechanisms have been described for persistent infections with Helicobacter pylori infection, streptococci, staphylococci, and yersinia [7,91].

In autoimmune type-B gastritis molecular mimicry between Helicobacter pylori and lipopolysaccharide (LPS) and anti-Lewis antibodies has been described [92,93]. In this regard, positivity of autologous serum skin test has been associated with Helicobacter pylori infection in chronic urticaria [94,95]. Interestingly, in some but not all patients with chronic urticaria autologous serum skin test became negative after Helicobacter eradication (own unpublished observation). Moreover, recent data found a significant difference in the prevalence of Helicobacter pylori infection between autoimmune urticaria with and without thyroid autoimmunity (90.9% vs. 46.7%; P = 0.02). Autoimmune thyroiditis was associated with cag A+ Helicobacter pylori strains [96]. Thus, in immunologically predisposed individuals, infection with Helicobacter pylori may result in the manifestation of a latent autoimmune pathomechanism.

Regarding molecular mimicry for other pathogens, it may be of note that infection with yersinia is linked to autoimmune thyroiditis [97]. Accordingly, persistence of streptococcal infection has been associated with autoimmunity [98,99]. An example for this phenomenon is the molecular mimicry between haemolytic streptococcus group A antigens and host proteins, which has been studied in detailed and lead to autoimmune reactions both humoral and cell mediated causing rheumatic fever and rheumatic heart disease [100].

## Conclusion

Controversy surrounds the role of occult infection in the pathogenesis of urticaria subtypes. At least in spontaneous urticaria the role is well established for acute urticaria but due to the limited nature of acute urticaria this does not result in a specific management. Although in chronic urticaria randomized controlled trials are lacking there is increasing evidence that persistent infections are important triggering factors. This is particularly the case for infection with Helicobacter pylori. Summarizing available studies that have proven Helicobacter eradication the remission/improvement rate of chronic urticaria is nearly doubled compared to untreated Helicobacter-positive or Helicobacter-negative controls (p < 0.001). Nevertheless, the pathogenesis of chronic urticaria in an individual patient may be multifactorial and not only infectious. In this regard, it will be interesting to elucidate the potential link between persistent infection and the development of autoimmune mechanisms in chronic urticaria which is also under investigation for example in autoimmune thrombocytopenic thrombopenia.

Given the marginally effective and sometimes risky medical therapy (e. g. immunosuppressant drugs) available for chronic urticaria, a systematic and thorough approach to identify a treatable cause in difficult chronic urticaria patients is warranted. In contrast to the autoimmune mechanisms in chronic urticaria against which no specific treatment strategy has been developed so far, infections are easy to treat and therefore a careful search for at least infections with Helicobacter pylori, streptococci, but perhaps also with staphylococci, and yersinia should be included in the diagnostic work-up in severely affected patients. Besides a careful history for infections (particularly gastro-intestinal, dental, ENT) our reliable routine diagnostic work-includes i) differential blood count and C-reactive protein, ii) Helicobacter pylori monoclonal stool antigen test, and iii) serology for streptococci (antistreptolysin, antiDNase B), staphylococci (antistaphylolysin), yersinia (IgA, IgG, immunoblot). If an infection is identified, it should be appropriately treated and it should be checked whether eradication has been achieved.

Future research will have to clarify why some people are more susceptible to developing urticaria and/or autoreactivity following a particular infection than others.

## Competing interests

The authors declare that they have no competing interests.

## Authors' contributions

BW wrote the manuscript. The review is based upon the clinical and scientific experience of BW, UR, DW and AK in the management of patients with urticaria. All authors read and approved the final manuscript.
